# Virtual chromoendoscopy with linked color imaging versus dye-chromoendoscopy in the surveillance of patients with long-standing colonic inflammatory bowel disease

**DOI:** 10.1093/ecco-jcc/jjag085

**Published:** 2026-06-19

**Authors:** Roberto Gabbiadini, Gianluca Franchellucci, Francesco Minini, Arianna Dal Buono, Giuseppe Privitera, Marco Spadaccini, Giulia Migliorisi, Lorenzo Petronio, Valeria Farinola, Antonio Di Martino, Cristina Bezzio, Cesare Hassan, Alessandro Repici, Alessandro Armuzzi

**Affiliations:** IBD Center, Department of Gastroenterology, IRCCS Humanitas Research Hospital, Via Manzoni 56, Rozzano, Milan 20089, Italy; Endoscopy Unit, Department of Gastroenterology, IRCCS Humanitas Research Hospital, Via Manzoni 56, Rozzano, Milan 20089, Italy; Endoscopy Unit, Department of Gastroenterology, IRCCS Humanitas Research Hospital, Via Manzoni 56, Rozzano, Milan 20089, Italy; Department of Biomedical Sciences, Humanitas University, Via Rita Levi Montalcini 4, Pieve Emanuele, Milan 20072, Italy; IBD Center, Department of Gastroenterology, IRCCS Humanitas Research Hospital, Via Manzoni 56, Rozzano, Milan 20089, Italy; IBD Center, Department of Gastroenterology, IRCCS Humanitas Research Hospital, Via Manzoni 56, Rozzano, Milan 20089, Italy; Department of Biomedical Sciences, Humanitas University, Via Rita Levi Montalcini 4, Pieve Emanuele, Milan 20072, Italy; Endoscopy Unit, Department of Gastroenterology, IRCCS Humanitas Research Hospital, Via Manzoni 56, Rozzano, Milan 20089, Italy; Department of Biomedical Sciences, Humanitas University, Via Rita Levi Montalcini 4, Pieve Emanuele, Milan 20072, Italy; IBD Center, Department of Gastroenterology, IRCCS Humanitas Research Hospital, Via Manzoni 56, Rozzano, Milan 20089, Italy; Department of Biomedical Sciences, Humanitas University, Via Rita Levi Montalcini 4, Pieve Emanuele, Milan 20072, Italy; IBD Center, Department of Gastroenterology, IRCCS Humanitas Research Hospital, Via Manzoni 56, Rozzano, Milan 20089, Italy; Department of Biomedical Sciences, Humanitas University, Via Rita Levi Montalcini 4, Pieve Emanuele, Milan 20072, Italy; IBD Center, Department of Gastroenterology, IRCCS Humanitas Research Hospital, Via Manzoni 56, Rozzano, Milan 20089, Italy; Department of Biomedical Sciences, Humanitas University, Via Rita Levi Montalcini 4, Pieve Emanuele, Milan 20072, Italy; IBD Center, Department of Gastroenterology, IRCCS Humanitas Research Hospital, Via Manzoni 56, Rozzano, Milan 20089, Italy; Department of Biomedical Sciences, Humanitas University, Via Rita Levi Montalcini 4, Pieve Emanuele, Milan 20072, Italy; IBD Center, Department of Gastroenterology, IRCCS Humanitas Research Hospital, Via Manzoni 56, Rozzano, Milan 20089, Italy; Department of Biomedical Sciences, Humanitas University, Via Rita Levi Montalcini 4, Pieve Emanuele, Milan 20072, Italy; Endoscopy Unit, Department of Gastroenterology, IRCCS Humanitas Research Hospital, Via Manzoni 56, Rozzano, Milan 20089, Italy; Department of Biomedical Sciences, Humanitas University, Via Rita Levi Montalcini 4, Pieve Emanuele, Milan 20072, Italy; Endoscopy Unit, Department of Gastroenterology, IRCCS Humanitas Research Hospital, Via Manzoni 56, Rozzano, Milan 20089, Italy; Department of Biomedical Sciences, Humanitas University, Via Rita Levi Montalcini 4, Pieve Emanuele, Milan 20072, Italy; IBD Center, Department of Gastroenterology, IRCCS Humanitas Research Hospital, Via Manzoni 56, Rozzano, Milan 20089, Italy; Department of Biomedical Sciences, Humanitas University, Via Rita Levi Montalcini 4, Pieve Emanuele, Milan 20072, Italy

**Keywords:** Crohn’s disease, ulcerative colitis, surveillance colonoscopy, colonic lesions, dysplasia detection

## Abstract

**Background and aims:**

Surveillance colonoscopy programmes in inflammatory bowel disease (IBD) are associated with reduced colorectal cancer (CRC) incidence. This study aims to compare the performance of virtual chromoendoscopy (VCE) using linked color imaging (LCI) and dye-chromoendoscopy (DCE) with methylene blue for the detection of colonic neoplastic lesions during surveillance colonoscopy in patients with IBD.

**Methods:**

This single-center retrospective cohort study included patients with colonic IBD undergoing CRC surveillance colonoscopy between June 2018 and December 2024. The primary outcome was the total neoplastic lesions detected in each group. The secondary outcome was the neoplastic lesions detection rate. Propensity-score matching and multivariable Poisson and logistic regression were used to adjust for baseline differences and identify independent predictors.

**Results:**

311 patients were included (112 DCE, 199 LCI). The mean number of neoplastic lesions identified per colonoscopy was 0.116 with DCE and 0.085 with LCI (*P* = .472). Similarly, the neoplasia detection rate was comparable between techniques (8.03% for DCE vs 7.53% for LCI; odds ratio [OR] 1.07, 95% confidence interval [CI], 0.45-2.54, *P* = .874). After 1:1 propensity score matching, the chromoendoscopy modality (DCE vs LCI) was not associated with the total number of neoplastic lesions (IRR 0.77; 95% CI, 0.34-1.78; *P* = .543) and with the neoplastic lesions detection rate (OR 0.58; 95% CI, 0.21-1.65; *P* = .310).

**Conclusions:**

LCI-based VCE provides a neoplastic yield comparable to traditional DCE in patients with long-standing colonic IBD undergoing surveillance colonoscopy. As with other VCE modalities, LCI may be adopted as an alternative to DCE for dysplasia surveillance in IBD.

## 1. Introduction

Persistent inflammation of the intestinal mucosa is considered the primary trigger for the development of colorectal dysplasia and colorectal cancer (CRC) in inflammatory bowel disease (IBD).^1^ Patients with long-standing colonic IBD are at increased risk of developing CRC,^2–5^ with a risk 1.5-2 times higher than in the general population.^1^ Surveillance colonoscopy programmes in IBD are associated with reduced CRC incidence^6^ and are recommended by many international guidelines, generally beginning 8-10 years after disease onset.^7–9^

Advances in endoscopic technology, such as high-definition (HD) colonoscopes, provide higher-resolution images for detecting colonic dysplasia compared with previous standard-definition colonoscopies.^7^^,^^10^ Furthermore, dye-chromoendoscopy (DCE) in conjunction with HD colonoscopy can enhance identification of colonic lesions and has been recommended as the preferred modality for endoscopic surveillance, albeit with low-grade evidence.^10^^,^^11^ Despite this, uptake of DCE for dysplasia surveillance in IBD remains low, with approximately 26.5%-44.6% of the endoscopists using it routinely, primarily due to longer procedure time and cost.^12^^,^^13^ Dye-less virtual chromoendoscopy (VCE) has emerged as an alternative contrast-enhancement system that instantly improves mucosal details and vascularity by means of optical filters or pre-/post-processing image technology incorporated in the endoscope, thereby saving time and costs during colonoscopy.^10^^,^^14^^,^^15^ Various VCE technologies are available, including narrow band imaging (NBI, Olympus, Japan), flexible imaging color enhancement (FICE, Fujifilm, Japan), i-SCAN and i-SCAN OE (Pentax, Japan), blue light imaging (BLI, Fujifilm, Japan), and linked color imaging (LCI, Fujifilm, Japan).^10^^,^^14^ A meta-analysis of 11 randomized controlled trials showed similar dysplasia detection between DCE and VCE^16^ and most scientific societies support VCE as a suitable alternative to DCE.^7^^,^^17^^,^^18^ However, most VCE studies have evaluated the performance of NBI and i-SCAN technologies.^16^^,^^19^ LCI is a more recent VCE modality that produces an amplified contrast of the red color spectrum of the gastro-intestinal mucosa compared to white light endoscopy (WLE), enhancing vascularity and reinforcing contrast for the detection of colonic lesions.^20^^,^^21^ As a result, LCI makes lesions appear more reddish and the adjacent mucosa more whitish.^10^^,^^21^^,^^22^ LCI has been associated with a higher number of adenomas detected per patient in non-IBD population.^23^ However, data on the performance of LCI for detecting dysplasia specifically among IBD patients undergoing surveillance remain scarce.^24^^,^^25^

The aim of this study was to compare the performance of VCE using LCI and DCE in the detection of colonic neoplastic lesions during surveillance colonoscopy in IBD patients in a real-life setting.

## 2. Material and methods

### Study population

We conducted a single-center, retrospective, observational cohort study at IRCCS Humanitas Research Hospital (Rozzano, Milan, Italy) including subjects with colonic IBD undergoing CRC surveillance colonoscopy according to the European Crohn’s and Colitis Organization (ECCO) guidelines, with procedures performed at appropriate surveillance intervals. Patients were recruited between June 2018 and December 2024, the period during which ELUXEO 700 HD colonoscopes (Fujifilm, Japan) were available at our institution.

DCE with methylene blue was the standard surveillance modality in our center until the updated 2019 ESGE guidelines,^18^ after which LCI-based chromoendoscopy was gradually introduced and increasingly used in clinical practice. Inclusion criteria were: patients aged  ≥18 years with a long standing disease (8 years or more after onset of symptoms) and left-sided or extensive ulcerative colitis (UC), or long-standing ileocolonic or colonic Crohn’s disease (CD) involving at least one-third of the colon. In case of concomitant primary sclerosing cholangitis (PSC), surveillance colonoscopy was performed annually regardless of disease duration.

Exclusion criteria were: pregnancy, inadequate bowel preparation, active UC (defined as a Mayo endoscopic score > 1) extending > 20 cm from the anal verge; active CD with Simple Endoscopic Score of Crohn’s disease (SES-CD) ≥ 5; colonic stenosis or incomplete colonoscopy; coagulopathy precluding biopsy or polypectomy. Patients with a history of surgical resection were eligible, except those with subtotal or total colectomy. Bowel preparation was assessed using the Boston bowel preparation score (BBPS) and defined adequate if the total score was ≥ 6 with no segment < 2.

All colonoscopies were performed by 3 expert endoscopists in IBD field and were conducted using the same HD colonoscopes (ELUXEO 700 series video colonoscopes: EC-760 R, EC-760 ZP; Fujifilm, Japan).

All participating endoscopists had comparable levels of expertise and followed a standardized inspection protocol, including a targeted-only biopsy strategy, a careful mucosal inspection during withdrawal and segmental evaluation of the colon, without routinely using additional re-inspection techniques. No significant differences in training or technique were present among operators. DCE was performed with 0.04% methylene blue using a spray catheter through the accessory channel to stain each segment of the colon. For VCE, LCI was activated and maintained during the withdrawal. The location, size, and morphology of all the visible colonic alterations were recorded for both groups according to the SCENIC consensus.^26^ For each colonoscopy, we collected details such as bowel preparation quality, presence of post-inflammatory polyps, tubular appearance of the colon. Lesions were histologically defined as colitis associated dysplasia (low-grade or high-grade), adenocarcinoma, serrated lesions, and sporadic adenoma (located outside the colitis area). Demographic and clinical data were extracted from medical records including age, gender, smoking status, presence of PSC, family history of CRC, history of colonic lesions, disease duration, IBD type and extent, and therapy at the time of endoscopy.

### Outcomes

The primary outcome was the detection of total neoplastic lesions, defined as the mean number of neoplastic lesions detected per colonoscopy, including colitis-associated dysplasia, adenocarcinoma, sporadic adenoma, serrated lesions, by targeted biopsies or polypectomy with either DCE or LCI. The secondary outcome was the neoplastic lesions detection rate, defined as the proportion of colonoscopies with at least 1 neoplastic lesion over the total number of colonoscopies.^27^

### Statistical analysis

Continuous variables are reported as mean ± standard deviation (SD) or median and interquartile range (IQR), as appropriate, and were compared using paired Student’s *t*-test or Mann–Whitney before matching and Wilcoxon Signed-Rank test after matching. Categorical variables are presented as counts and percentages and were compared using the Chi-square and McNemar’s test before and after matching respectively. To account for baseline imbalances in key risk factors, a propensity-score model was built including disease duration, PSC, personal history of colonic lesions, first-degree family history of colorectal cancer, and extensive UC; 1:1 matching using a nearest-neighbor algorithm was then applied within a specified caliper distance, set to a width equal to 0.2 of the standard deviation of the logit of the propensity score, and covariate balance was evaluated using standardized mean differences. The primary outcome was analyzed using multivariable Poisson regression to estimate incidence rate ratios (IRRs) and 95% confidence intervals (CIs), while multivariable logistic regression was used to identify independent predictors of neoplastic lesions detection rate, expressed as odds ratios (ORs) with 95% CIs. Variables included in multivariable models were selected a priori based on clinical relevance. All tests were 2-sided and a *P-*value < .05 was considered statistically significant. Statistical analyses were conducted using Stata 18.

## 3. Results

**Figure 1.**
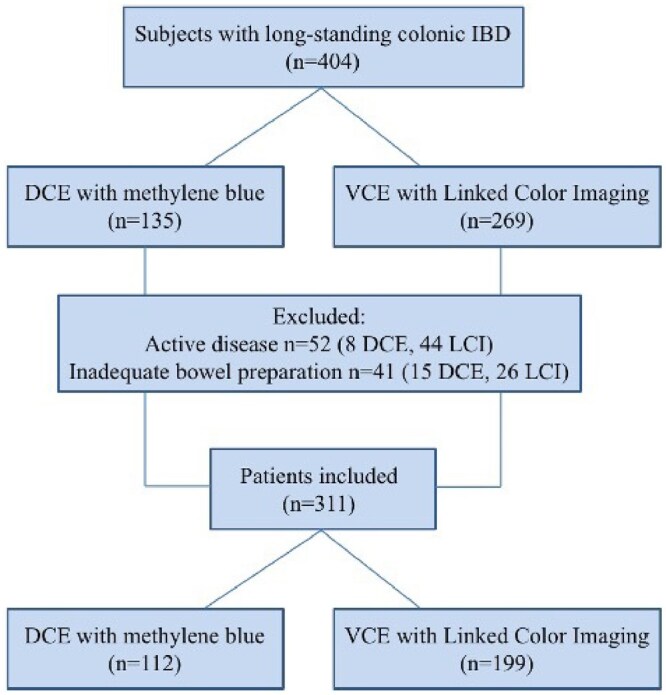
Flow chart of the study.

A total of 404 patients underwent surveillance colonoscopy during the study period. After exclusion of 93 patients because of inadequate bowel preparation or endoscopically active disease, 311 were included in the final analysis (112 DCE, 199 LCI). Figure 1 represent the study flow chart. The mean age of the cohort was 47.8 years, and 53.7% (*n* = 167) were male. Most patients had UC (83.3%, *n* = 259), while the remainder had colonic or ileocolonic CD. Among patients with UC, 72 (72.7%) had Mayo Endoscopic score of 0 in the DCE group and 114 (71.3%) in the LCI group (*P* = .797). The 2 chromoendoscopy techniques were evenly distributed among the participating endoscopists. Baseline characteristics of the study population, including both balanced and unbalanced variables between the 2 groups due to the retrospective study design, are presented in Table 1.

**Table 1.** Patients’ baseline characteristics.

	**Dye-chromoendoscopy (*n*** = **112)**	**Linked color imaging (*n*** = **199)**	*P*-value
**Age at colonoscopy, years (95% CI)**	48.19 (45.95-50.44)	47.50, (45.50-49.50)	.664
**Female (*n*, %)**	43 (38.39)	101 (50.75)	.036
**UC (*n*, %)**	99 (88.39)	160 (80.40)	.070
**Disease duration, years (95% CI)**	19.58 (17.95-21.20)	17.12 (15.84-18.39)	.021
**E2, UC (*n*, %)**	55 (49)	109 (54.77)	.337
**E3, UC (*n*, %)**	44 (39)	51 (26)	.012
**L2, CD (*n*, %)**	4 (3.5)	11 (5.5)	.440
**L3, CD (*n*, %)**	9 (8)	28 (14.1)	.115
**Smokers (*n*, %)**	15 (13.3)	32 (16.1)	.525
**Primary sclerosing cholangitis (*n*, %)**	12 (10.7)	7 (3.5)	.015
**Personal history of colonic lesions (*n*, %)**	16 (14.3)	13 (6.5)	.024
**1 degree family history of CRC (*n*, %)**	10 (8.9)	8 (4)	.082
**Tubular appearance of the colon (*n*, %)**	7 (6.2)	19 (9.5)	.313
**Pseudopolyps (*n*, %)**	20 (17.8)	34 (17.0)	.863
**No treatment (*n*, %)**	9 (8)	14 (7)	.746
**Mesalazine (*n*, %)**	83 (74)	123 (62)	.028
**Steroids (*n*, %)**	0	1 (0.5)	.454
**Azatioprine (*n*, %)**	0	1 (0.5)	.454
**Biological treatment (*n*, %)**	36 (32.1)	88 (44)	.037
**JAKi (*n*, %)**	1 (0.9)	13 (6.5)	.021
**S1PRMs (*n*, %)**	0 (0)	2 (1)	.287
**Multiple therapy (*n*, %)**	24 (21.4)	45 (22.6)	.809

Abbreviations: CD, Crohn’s disease; CI, confidence interval; CRC, colorectal cancer; JAKi, Janus kinase inhibitors; S1PRMs, sphingosine-1-phosphate receptor modulators; UC, ulcerative colitis.

**Figure 2.**
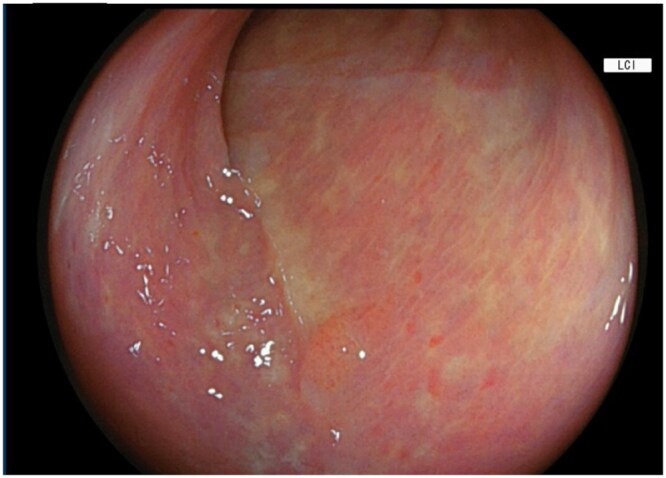
Low-grade colitis-associated dysplasia in the sigmoid colon detected with linked color imaging.

Overall, 30 neoplastic lesions were identified, all of which were endoscopically visible. The spectrum of neoplastic lesions was similar between the groups and is reported in Table 2. There was no significant difference in the mean number of neoplastic lesions per colonoscopy between DCE and LCI (0.116 vs 0.085, respectively; *P* = .472). Similarly, the overall neoplastic lesion detection rate did not differ significantly between DCE and LCI (8.03% vs 7.53%; OR 1.07, 95% CI, 0.45-2.54, *P* = .874). Figure 2 shows a colitis-associated dysplasia detected with LCI.

**Table 2.** Distribution and characteristics of neoplastic lesions in the dye-chromoendoscopy (DCE) and linked color imaging (LCI) groups.

Lesion type	**DCE (*n*** = **13)**	**LCI (*n*** = **17)**
**Colitis-associated dysplasia**	5	7
** Polypoid**	2 (2 LGD), 5-8 mm	5 (5 LGD), 3-15 mm
** Non-polypoid**	3 (2 LGD, 1 HGD), 5-15 mm	2 (2 LGD), 5 mm
**IBD-associated CRC**	1	1
**Sporadic adenomas (LGD)**	7 (7 LGD), 3-8 mm	5 (5 LGD), 3-7 mm
**Serrated lesions**	0	4, 4-5 mm

Abbreviations: CRC, colorectal cancer; HGD, high grade dysplasia; LGD, low grade dysplasia.

After the 1:1 propensity-score matching, 208 patients (104 DCE and 104 LCI) were available for analysis. In the matched cohort, baseline characteristics were balanced across groups, with substantial reduction in standardized mean differences and overall bias, as shown in Tables S1-S3 and Figure S1 (Love plot), and summarized in Table 3. Small residual differences persisted only for a few variables (eg, prior colonic surgery and tubular appearance of the colon), while the distributions of the main clinical and endoscopic risk factors were comparable between the 2 arms.

**Table 3.** Baseline characteristics after 1:1 propensity score matching on disease duration, primary sclerosing cholangitis, personal history of colonic dysplasia, first-degree family history of CRC, and extensive colitis.

	**Dye-chromoendoscopy (*n*** = **104)**	**Linked color imaging (*n*** = **104)**	*P*-value
**Age at colonoscopy, years (95% CI)**	47.22	50.46	.081
**Female (*n*, %)**	64	40	.070
**UC (*n*, %)**	91	88	.548
**Disease Duration, years (95% CI)**	18.60	19.41	.504
**E2, UC (*n*, %)**	52	50	.781
**E3, UC (*n*, %)**	39	38	.886
**L2, CD (*n*, %)**	4	6	.517
**L3, CD (*n*, %)**	9	10	.810
**Smokers (*n*, %)**	14	9	.269
**Primary sclerosing cholangitis (*n*, %)**	10	7	.448
**Personal history of colonic lesions (*n*, %)**	11	12	.825
**1° degree family history of CRC (*n*, %)**	9	8	.800
**Tubular appearance of the colon (*n*, %)**	6	15	.038
**Pseudopolyps (*n*, %)**	19	18	.856
**No treatment (*n*, %)**	8	9	.800
**Mesalazine (*n*, %)**	77	71	.358
**Steroids (*n*, %)**	0	1	.318
**Azatioprine (*n*, %)**	12	4	.037
**Biological treatment (*n*, %)**	32	36	.554
**JAKi (*n*, %)**	1	7	.031
**S1PRMs (*n*, %)**	0	1	.316
**Multiple therapy (*n*, %)**	20	22	.730

Abbreviations: CD, Crohn’s disease; CRC, colorectal cancer; JAKi, Janus kinase inhibitors; S1PRMs, sphingosine-1-phosphate receptor modulators; UC, ulcerative colitis.

At univariate analysis disease duration and first-degree family history of CRC were significative related to the number of neoplastic lesions. Multivariable Poisson regression was used to identify predictors of the total number of neoplastic lesions (Table 4). Longer disease duration was independently associated with a higher lesion count (incidence rate ratio [IRR] 1.05 per year; 95% CI, 1.01-1.10; *P* = .009), and a first-degree family history of CRC was also an independent predictor (IRR 3.64; 95% CI, 1.34-9.93; *P* = .012). A personal history of colonic lesions showed a non-significant trend toward a higher lesion count (IRR 2.29; 95% CI, 0.75-7.07; *P* = .147). Chromoendoscopy modality (DCE vs LCI) was not associated with the total number of neoplastic lesions (IRR 0.77; 95% CI, 0.34-1.78; *P* = .543).

**Table 4.** Poisson regression model for predictors of total neoplastic lesions.

	Univariate			Multivariate		
Variable	IRR	*P*-value	95% CI	IRR	*P*-value	95% CI
**1 degree family history of CRC**	3.97	.004	1.563-10.05	3.64	.012	1.336-9.932
**Disease duration**	1.06	.002	1.024-1.106	1.05	.009	1.014-1.101
**Personal history of colonic lesions**	1.69	.338	0.577-4.977	2.29	.147	0.746-7.070
**Extensive colitis**	0.47	.138	0.175-1.27	0.70	.508	0.248-1.993
**Chromoendoscopy type**	0 .77	.533	0.337-1.754	0.77	.543	0.335-1.779

Abbreviations: CI, confidence interval; CRC, colorectal cancer; IRR, incidence rate ratio.

In the logistic regression model exploring factors associated with the neoplastic lesions detection rate (Table 5), longer disease duration (OR 1.06 per year; 95% CI, 1.01-1.12; *P* = .027) and a first-degree family history of CRC (OR 4.43; 95% CI, 1.13-17.38; *P* = .032) emerged as independent predictors. A personal history of colonic lesions was associated with a numerically higher, but borderline non-significant, neoplastic lesions detection rate (OR 3.56; 95% CI, 0.98-12.90; *P* = .053). Again, the type of chromoendoscopy (DCE vs LCI) was not independently associated with the neoplastic lesions detection rate (OR 0.58; 95% CI, 0.21-1.65; *P* = .310).

**Table 5.** Logistic regression model for predictors of neoplastic lesions detection rate.

	Univariate			Multivariate		
Variable	OR	*P*-value	95% CI	OR	*P*-value	95% CI
**1 degree family history of CRC**	3.89	.033	1.119-13.520	4.43	.032	1.133-17.378
**Disease duration**	1.06	.014	1.012-1.119	1.06	.027	1.007-1.119
**Personal history of colonic lesions**	2.57	.125	0.768-8.606	3.56	.053	0.984-12.901
**Extensive colitis**	0.63	.399	0.216-1.842	0.91	.879	0.289-2.894
**Chromoendoscopy type**	0.610	.328	0.227-1.641	0.58	.310	0.207-1.650

Abbreviations: CI, confidence interval; CRC, colorectal cancer; OR, odds ratio.

In addition, to account for potential chronological and treatment-related confounding, sensitivity analyses including calendar year of colonoscopy and biological therapy (anti-TNF vs non anti-TNF biologics) in separate propensity score models were performed. Despite smaller matched cohorts (79 patients per group for calendar year of colonoscopy and 37 patients per group for biological therapy), results remained consistent with the primary analysis, with no significant differences between DCE and LCI (Tables S4, S5, S7, and S8).

A further sensitivity analysis restricted to colitis-associated dysplasia is reported in Table S6, yielding consistent findings for the type of chromoendoscopy despite the limited number of events.

Finally, with respect to withdrawal time between the 2 types of chromoendoscopy, DCE was associated with a longer duration than LCI (17.47 min [95% CI, 16.32-17.23] vs 14.21 min [95% CI, 13.38-15.04], respectively; *P* < .0001).

## 4. Discussion

DCE has been recommended by the SCENIC consensus as the preferred technique when performing surveillance colonoscopy in IBD, although it has been observed a low adherence by physicians, mainly due to the longer duration of the examination.^12^^,^^13^ Therefore, huge efforts were carried out to assess if the user-friendly dye-less VCE could become a valid alternative to DCE for this purpose. To date, various types of VCE have been investigated,^27–30^ although most data derived from NBI and i-SCAN. LCI is a VCE that has been shown to increase the adenoma detection rate and to reduce the miss rate of neoplastic lesions in a non-IBD setting.^20^^,^^31^^,^^32^ The performance of LCI in the CRC surveillance in IBD is unclear and our study adds specifically to the limited evidence base for LCI, which has so far been restricted largely to case reports.^24^^,^^25^ In this retrospective study involving subjects with longstanding colonic IBD, we did not find a significantly different neoplasia diagnostic yield between DCE using methylene blue and VCE with LCI. We observed a similar detection of total neoplastic lesions and also a similar neoplastic lesions detection rate in both groups, even after adjusting for independent risk factors in the multivariate analysis. By applying LCI-based VCE in a real-world setting, our study provides validation of its equivalence to dye-based chromoendoscopy, both of which are currently endorsed by ECCO guidelines for dysplasia surveillance in IBD.^7^

This is slightly in contrast with a recent network meta-analysis and the new British Society of Gastroenterology (BSG) guidelines, which recommend the use of HD DCE, as it has demonstrated a modest benefit compared with HD WLE.^11^^,^^33^ However, a direct comparison between DCE and VCE has not been performed in the meta-analysis, which stated that there was no evidence supporting any of the other modalities as alternatives due to very low-certainty evidence, rather than any demonstrated inferiority.^11^

Moreover, as also demonstrated in our study, VCE is known to be a less time-consuming surveillance alternative to DCE,^19^ which may improve feasibility and uptake in routine practice.

Corroborating these findings, in our multivariable analyses, longer disease duration and a first-degree family history of CRC were independently associated with a higher number of neoplastic lesions. These results are consistent with the well-established role of cumulative inflammatory burden and familial predisposition as key drivers of colitis-associated neoplasia. The effect sizes observed for these factors reinforce the importance of investigating new risk-stratified surveillance programs.^34^

Several methodological aspects of our study merit consideration. The analysis was conducted in a high-volume IBD center using a uniform HD endoscopic platform. Although the single-center design may limit external validity, it also represents a methodological strength, as it ensures procedural consistency, standardized technique, and uniform application of surveillance protocols.

Furthermore, we attempted to mitigate baseline imbalances between groups through propensity-score matching on key risk factors (disease duration, PSC, personal and family history of neoplasia, and extensive colitis) and through multivariable regression models. After matching, the distribution of major risk factors was well balanced, and the absence of a signal favoring either modality remained robust in adjusted analyses.

Nonetheless, several limitations should be acknowledged. First, the retrospective and non-randomized design inherently carries a risk of residual confounding and selection bias. While randomized controlled trials remain the gold standard for evaluating interventions, it should be noted that blinding the endoscopist to the type of chromoendoscopy is inherently impossible. In this context, our propensity score-matched analysis may better capture real-world practice, as endoscopists were unaware of study participation and conducted surveillance according to routine clinical behavior, thereby minimizing performance and observation biases.

On the other hand, all procedures were performed by expert endoscopists, which may limit the generalizability of our findings to lower-volume settings or to general gastroenterology practice.

Second, LCI was routinely adopted in our center following the updated ESGE guidelines, and despite the performance of a sensitivity analysis adjusting for calendar period, as well as consistent exclusion of active disease and inadequate preparation throughout the study period, unmeasured temporal trends cannot be fully excluded. Third, the overall number of neoplastic lesions was relatively small compared to other RCTs.^27^ This difference may be partially explained by the disparities in the population characteristics (ie, lower percentage of PSC and lower age at colonoscopy) and the study period, reflecting a more “modern” risk profile characterized by contemporary IBD management strategies, including a treat-to-target approach with a low threshold for acceptable mucosal inflammation.^35^ Differently from our study that was conducted in recent years (2018-2024), the RCTs have been carried out up to 10 years earlier (2008-2013)^27^ when the risk of CRC may have been higher.^36^ While this is understandable in contemporary surveillance cohorts, it limits statistical power and suggests that the study may be underpowered to detect small differences between the 2 chromoendoscopy techniques.

In conclusion, our real-world data show no evidence of a difference in neoplastic yield between LCI-based VCE and traditional DCE in patients with long-standing colonic IBD undergoing surveillance colonoscopy. To our knowledge, this is the first study to specifically evaluate LCI in the setting of surveillance for colonic IBD. Within HD endoscopic systems and structured surveillance programs, the choice of chromoendoscopy modality appears less critical than established risk factors such as disease duration and family history of colorectal cancer.

Future research should focus on adequately powered, prospective multicenter studies or randomized trials directly comparing LCI and DCE in IBD surveillance to confirm these findings.

## Supplementary Material

jjag085_Supplementary_Data

## Data Availability

The data underlying this article will be shared on reasonable request to the corresponding author.
