# Supplementary material for: Virtual chromoendoscopy with linked color imaging versus dye-chromoendoscopy in the surveillance of patients with long-standing colonic inflammatory bowel disease
Source: J Crohns Colitis. 2026 Jun 19;20(6):jjag085. doi: 10.1093/ecco-jcc/jjag085 (PMC13278836; doi:10.1093/ecco-jcc/jjag085)

**Supplementary Table 1.** Balance of Baseline Covariates Before and After Propensity Score Matching

| **Variable** | **Sample** | **Metilene blu**  **LCI** | **%bias** | **% reduce Bias** | **t-test**  **t p>\|t\|** | **V(T)/VC** |
| --- | --- | --- | --- | --- | --- | --- |
| Disease Duration | Unmatched  Matched | 19.58 17.12  18.59 19.41 | 27.6  -9.2 | 66.8 | 2.32 0.021  -0.67 0.505 | 0.90  0.65 |
| Primary sclerosing cholangitis | Unmatched  Matched | 0.10 0.04  0.10  0.07 | 28.2  11.3 | 59.9 | 2.56 0.011  0.76 0.450 | n.a |
| History of Colonic Lesion | Unmatched  Matched | 0.14  0.07  0.11  0.12 | 20.0  3.9 | 80.4 | 2.27 0.024  -0.22 0.826 | n.a |
| 1° degree family history of CRC | Unmatched  Matched | 0.09  0.04  0.09  0.08 | 25.5  -3.2 | 87.6 | 1.78 0.076  0.25 0.801 | n.a |
| E3, Ulcerative Colitis | Unmatched  Matched | 0.39  0.26  0.38  0.37 | 29.4  2.1 | 93.0 | 2.53 0.012  0.14 0.886 | n.a |

**Supplementary Table 2:** Overall Match Quality: Comparison of Unmatched and Propensity Score–Matched Samples

| **Sample** | **Ps R2** | **LR chi2** | **p> chi2** | **MeanBias** | **MedBias** | **B** | **% var** |
| --- | --- | --- | --- | --- | --- | --- | --- |
| Unmatched | 0.064 | 25.84 | 0.000 | 26.1 | 27.6 | 61.0 | 0 |
| Matched | 0.003 | 1.00 | 0.963 | 5.9 | 3.9 | 13.8 | 100 |

**Supplementary Table 3:** Standardized mean differences (SMD) and reduction in imbalance for selected baseline variables before and after matching

| **Variable** | **Cohort status** | **SMD** | **% reduction** |
| --- | --- | --- | --- |
| Disease duration (years) | Unmatched | 0.276 | 45.2 |
|  | Matched | 0.151 |  |
| Primary sclerosing cholangitis | Unmatched | 0.282 | 75.2 |
|  | Matched | -0.070 |  |
| History of Colonic Lesion | Unmatched | 0.255 | 88.5 |
|  | Matched | 0.029 |  |
| 1° degree family history of CRC | Unmatched | 0.200 | 81.8 |
|  | Matched | 0.036 |  |
| E3, ulcerative colitis | Unmatched | 0.294 | 60.8 |
|  | Matched | -0.115 |  |

**Supplementary Table 4:** Poisson regression model for predictors of total neoplastic lesions in the sensitivity analysis incorporating calendar year into the propensity score model.

|  | **Univariate** | | | **Multivariate** | | |
| --- | --- | --- | --- | --- | --- | --- |
| **Variable** | **IRR** | **p-value** | **95% CI** | **IRR** | **p-value** | **95% CI** |
| 1° degree family history of CRC | 11.5 | < 0.001 | 3.18- 41.96 | 6.89 | 0.009 | 1.635-29.045 |
| Disease Duration | 1.07 | 0.003 | 1.024- 1.12 | 1.05 | 0.056 | 0.999- 1.113 |
| Personal history of colonic lesions | 2.03 | 0.358 | 0.449- 9.15 | 2.05 | 0.369 | 0.428- 9.850 |
| Extensive Colitis | 0.56 | 0.381 | 0.155- 2.041 | 0.88 | 0.847 | 0.231-3.328 |
| Chromoendoscopy Type | 1 .17 | 0.782 | 0.392- 3.47 | 1.06 | 0.925 | 0.335-3.330 |
| 2018-2019 vs 2020-2024 | 1.14 | 0.832 | 0.35-3.69 | 1.02 | 0.973 | 0.292- 3.575 |

IRR: Incidence Rate Ratio; CI: Confidence Interval; CRC: colorectal cancer.

**Supplementary Table 5.** Logistic regression model for predictors of neoplastic lesion detection rate in the sensitivity analysis incorporating calendar year into the propensity score model.

|  | **Univariate** | | | **Multivariate** | | |
| --- | --- | --- | --- | --- | --- | --- |
| **Variable** | **OR** | **p-value** | **95% CI** | **OR** | **p-value** | **95% CI** |
| 1° degree family history of CRC | 3.89 | 0.033 | 1.119- 13.520 | 4.72 | 0.242 | 0.351- 63.41 |
| Disease Duration | 1.06 | 0.014 | 1.012- 1.119 | 1.06 | 0.096 | 0.989-1.138 |
| Personal history of colonic lesions | 2.57 | 0.125 | 0.768- 8.606 | 3.52 | 0.161 | 0.607- 20.46 |
| Extensive Colitis | 0.63 | 0.399 | 0.216- 1.842 | 1.27 | 0.758 | 0.278-5.80 |
| Chromoendoscopy Type | 0.610 | 0.328 | 0.227- 1.641 | 0.77 | 0.712 | 0.178-3.06 |
| 2018-2019 vs 2020-2024 | 1.010 | 0.989 | 0.242- 4.20 | 0.75 | 0.723 | 0.159-3.57 |

OR: Odds Ratio; CI: Confidence Interval; CRC: colorectal cancer.

**Supplementary Table 6**: Logistic regression model for predictors of colitis-associated dysplasia

detection rate.

|  | **Univariate** | | | **Multivariate** | | |
| --- | --- | --- | --- | --- | --- | --- |
| **Variable** | **OR** | **p-value** | **95% CI** | **OR** | **p-value** | **95% CI** |
| 1° degree family history of CRC | 1.429 | 0.743 | 0.169- 12.16 | 2.16 | 0.504 | 0.22-20.92 |
| Disease Duration | 1.02 | 0.464 | 0.957- 1.10 | 1.03 | 0.425 | 0.956- 1.11 |
| Personal history of colonic lesions | 4.47 | 0.044 | 1.04- 19.28 | 4.90 | 0.040 | 1.08-22.27 |
| Extensive Colitis | 1.38 | 0.638 | 0.359-5.305 | 1.58 | 0.528 | 0.377-6.69 |
| Chromoendoscopy Type | 0.792 | 0.734 | 0.158- 1.11 | 0.76 | 0.704 | 0.195-3.01 |

OR: Odds Ratio; CI: Confidence Interval; CRC: colorectal cancer.

**Supplementary Table 7**: Poisson regression model for predictors of total neoplastic lesions in the sensitivity analysis incorporating biological therapy (anti-TNF vs not anti-TNF) into the propensity score model.

|  | **Univariate** | | | **Multivariate** | | |
| --- | --- | --- | --- | --- | --- | --- |
| **Variable** | **IRR** | **p-value** | **95% CI** | **IRR** | **p-value** | **95% CI** |
| Disease Duration | 1.04 | 0.200 | 0.97-1.12 | 1.03 | 0.343 | 0.96- 1.12 |
| Personal history of colonic lesions | 3.84 | 0.065 | 0.92-16.06 | 2.12 | 0.370 | 0.408-11.10 |
| Extensive Colitis | 2.07 | 0.319 | 0.49-8.6 | 1.56 | 0.574 | 0.329- 7.41 |
| Chromoendoscopy Type | 3 | 0.178 | 0.605-14.86 | 2.43 | 0.298 | 0.456-12.96 |
| Anti-TNF vs not anti-TNF biologics | 2.07 | 0.319 | 0.495-8.67 | 1.50 | 0.588 | 0.342-6.62 |

IRR: Incidence Rate Ratio; CI: Confidence Interval; TNF: Tumor Necrosis Factor

**Supplementary Table 8**: Logistic regression model for predictors of neoplastic lesion detection rate in the sensitivity analysis incorporating biological therapy (anti-TNF vs not anti-TNF) into the propensity score model.

|  | **Univariate** | | | **Multivariate** | | |
| --- | --- | --- | --- | --- | --- | --- |
| **Variable** | **OR** | **p-value** | **95% CI** | **OR** | **p-value** | **95% CI** |
| Disease Duration | 1.063 | 0.129 | 0.983-1.15 | 1.06 | 0.206 | 0.968-1.163 |
| Personal history of colonic lesions | 6.42 | 0.031 | 1.187-34.81 | 3.21 | 0.249 | 0.440-23.50 |
| Extensive Colitis | 3.48 | 0.153 | 0.63-19.25 | 1.24 | 0.782 | 0.258-6.04 |
| Chromoendoscopy Type | 2.73 | 0.249 | 0.49-15.09 | 2.25 | 0.387 | 0.357-14.17 |
| Anti-TNF vs not anti-TNF biologics | 1.74 | 0.487 | 0.362-8.42 | 1.04 | 0.958 | 0.176-6.25 |

OR: Odds Ratio; CI: Confidence Interval; TNF: Tumor Necrosis Factor

**Supplementary Figure 1.** Love plot showing standardized mean differences before and after propensity-score matching.


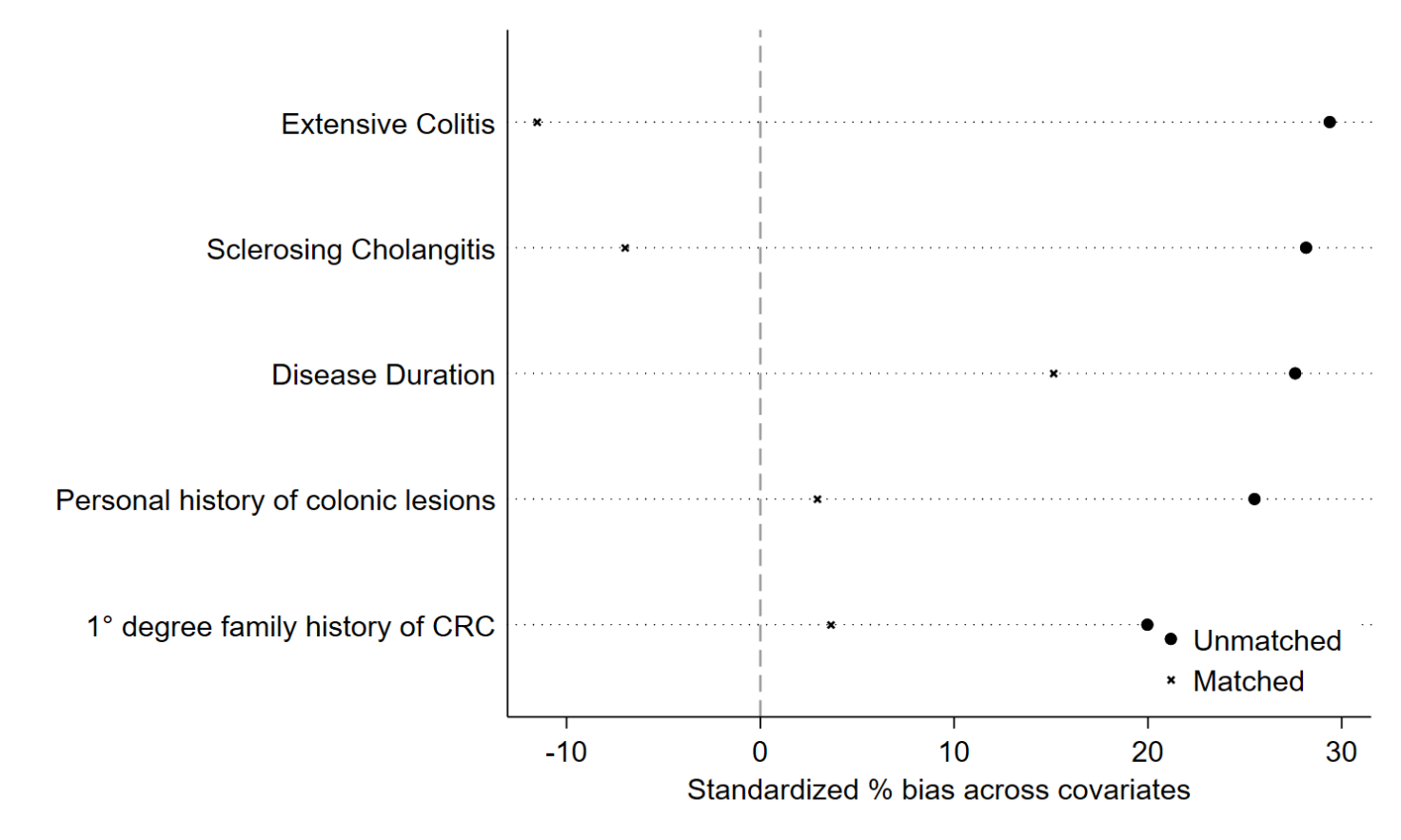

Supplement: jjag085_Supplementary_Data [file jjag085_supplementary_data.docx]
